# BRASD trial: biomechanical reposition techniques in anterior shoulder dislocation—a randomized multicenter clinical trial

**DOI:** 10.1186/s12245-023-00480-6

**Published:** 2023-02-24

**Authors:** David N. Baden, Martijn H. Roetman, Tom Boeije, Nieke Mullaart, Ralph Boden, Roderick M. Houwert, Marilyn Heng, Rolf H. H. Groenwold, Olivier A. J. van der Meijden

**Affiliations:** 1 Diakonessenhuis, Utrecht, The Netherlands; 2 Flevoziekenhuis, Almere, The Netherlands; 3 Dijklander Ziekenhuis, Hoorn, The Netherlands; 4 Bravis Ziekenhuis, Bergen op Zoom and Roosendaal, The Netherlands; 5 University Medical Center Utrecht, Utrecht, The Netherlands; 6 Massachusetts General Hospital, Boston, USA; 7 Department of Clinical Epidemiology, Leiden University Medical Center, Leiden, the Netherlands; 8 Albert Schweitzer Ziekenhuis, Dordrecht, The Netherlands

**Keywords:** Anterior shoulder dislocation, Biomechanical reduction techniques, Length-of-stay, Emergency department, Reduction rate, No medication

## Abstract

**Background:**

Biomechanical reduction techniques for shoulder dislocations have demonstrated high reduction success rates with a limited pain experience for the patient. We postulated that the combination of biomechanical reduction techniques with the shortest length of stay would also have the lowest pain experience and the highest first reduction success rate.

**Methods:**

A randomized multicenter clinical trial was performed to compare different biomechanical reduction techniques in treating anterior shoulder dislocations without the use of invasive pain relief. Patients who were able to perform adduction of the arm were randomly assigned to Cunningham, the modified Milch, and the scapular manipulation technique. Those who were not able to do so were randomly assigned to modified Milch and the scapular manipulation technique. Primary outcomes were emergency department length of stay and pain experienced during the reduction process, measured by the numeric pain rating scale. Secondary outcomes were reduction time, reduction success, use of analgesics or sedatives, and complications.

**Results:**

Three hundred eight patients were included, of whom 134 were in the adduction group. In both groups, no differences in emergency department length of stay and experienced pain were observed between the treatment arms. In the adduction group, the modified Milch technique had the highest first reduction success rates 52% (*p* = 0.016), within protocol 61% (*p* = 0.94), and with sedation in the ED 100% ( −). In the no-adduction group, the modified Milch was also the most successful primary reduction technique with 51% success (*p* = 0.040), within protocol 66% (*p* = 0.90), and with sedation in the ED 98% (*p* = 0.93). No complications were recorded in any of the techniques.

**Conclusion:**

A combination of biomechanical techniques resulted in a similar length of stay in the emergency department and showed similar pain scores with an overall high success rate of reduction. In both groups, the modified Milch had the highest first-reduction success rate.

**Trial registration:**

Netherlands Trial Register NTR5839—1 April 2016. Ethical committee Noord-Holland with the CCMO-number NL54173.094.15

**Supplementary Information:**

The online version contains supplementary material available at 10.1186/s12245-023-00480-6.

## Background

An anterior shoulder dislocation has a major impact on the patient and places high demands on emergency department (ED) facilities.

The dislocation is often very painful, and the primary treatment is a timely closed reduction. Pain is caused by muscle spasms in the rotator cuff, which also causes the biceps to contract, thus maintaining the dislocation. More than 50 reduction techniques have been described that can be grouped into traction-counter-traction techniques (TCT), leverage techniques, and biomechanical techniques [1, 2].

For the patient, as well as for the likelihood of reduction success, an important element is pain relief. Procedural sedation and analgesia (PSA) or intra-articular lidocaine (IAL) techniques require additional personnel and take time to set up, and PSA in particular extends emergency department stay considerably. These techniques also carry a risk of complications [3, 4]. Another possible method of pain relief is using a reduction technique that has no  influence or has a positive effect on the pain experience.

Previous randomized trials concerning shoulder reduction techniques, without the use of PSA or IAL, showed mixed results in success rate and pain relief [5, 6]. In traction-counter traction and leverage technique complications are described, and the application of more force seems to increase the risk of complications [7, 8].

In previous studies on shoulder dislocation, the primary outcome parameter was reduction success. Although initial success rates vary across studies, eventually almost all techniques appear to have a success rate of 80–90% [7, 9, 10]. Therefore, we considered emergency department length of stay (LOS) an alternative primary outcome measure which arguably better reflects the burden and safety for the patient, impacts the emergency room, and offers a different viewpoint.

The Cunningham technique (CH), modified Milch (MM), and scapular manipulation technique (SMT) are among the most frequently used biomechanical reduction techniques [11]. In contrast to traction-counter traction and leverage techniques, biomechanical techniques are primarily performed without invasive pain relief like PSA or IAL [5, 6, 12, 13]. To date, however, there are no studies directly comparing these three biomechanical techniques.

Therefore, the aim of this study was to compare these different biomechanical reduction techniques without the use of invasive pain relief regarding emergency department length of stay (LOS) and experienced pain. We hypothesized that the combination technique with reduced LOS would also have the lowest pain experience and the highest first reduction success rate.

## Methods

For a detailed description of the design of this study, we refer to the study protocol [1]. During the study, no changes were made to the study protocol. Approval for this study was obtained from the local ethics committee (CCMO-number NL54173.094.15) and registered in the Netherlands Trial Register (NTR5839). The Consort guidelines were followed (the CONSORT checklist is added as supplemental file 1).

### Inclusion

From August 1, 2016, to December 31, 2018, all patients who presented with an anterior shoulder dislocation at the emergency departments of four regional hospitals across the Netherlands were screened for enrolment. All involved hospitals are level 2 trauma centers with an annual ED volume of approximately 30,000 patients.

Eligible patients, with an anterior shoulder dislocation confirmed by radiograph, were included by the treating clinicians when aged ≥ 18 years old. In patients who suffered recurrent shoulder dislocations, minimal trauma (i.e., no fall or body-to-body contact), and a strong clinical suspicion of dislocation, the radiograph could be omitted. Patients were excluded if they suffered from multi-trauma, if the dislocation coincided with a fracture of the proximal humerus, or if the presence of the dislocation was more than 24 h. The latter is due to the possible increased risk of treatment failure as previously described and for comparison purposes with existing literature [5, 6, 14]. Written informed consent was obtained from all participants.

### Randomization and treatment allocation

The participants were classified by the treating physician based on the ability to perform adduction (active or passive) of the injured arm by touching the torso with the elbow. Adduction is an important part of the Cunningham technique, without adduction, this technique cannot be performed.

Within each of the groups (“adduction” versus “no adduction”), patients were then randomized with equal probability to the different treatment arms using a computer-generated block randomization, stratified by center, with a block size of 10. When a patient with a possible anterior shoulder dislocation entered the emergency department, the treating physician was given two blinded envelopes containing the randomized techniques. A green envelope was opened if adduction could be performed and a red envelope was opened if adduction could not be performed. The unused envelope was returned. The envelopes were numbered for each individual hospital in order to maintain randomization order. Patients and doctors could not be blinded to the individual techniques as this was technically impossible.

**Fig. 1:**
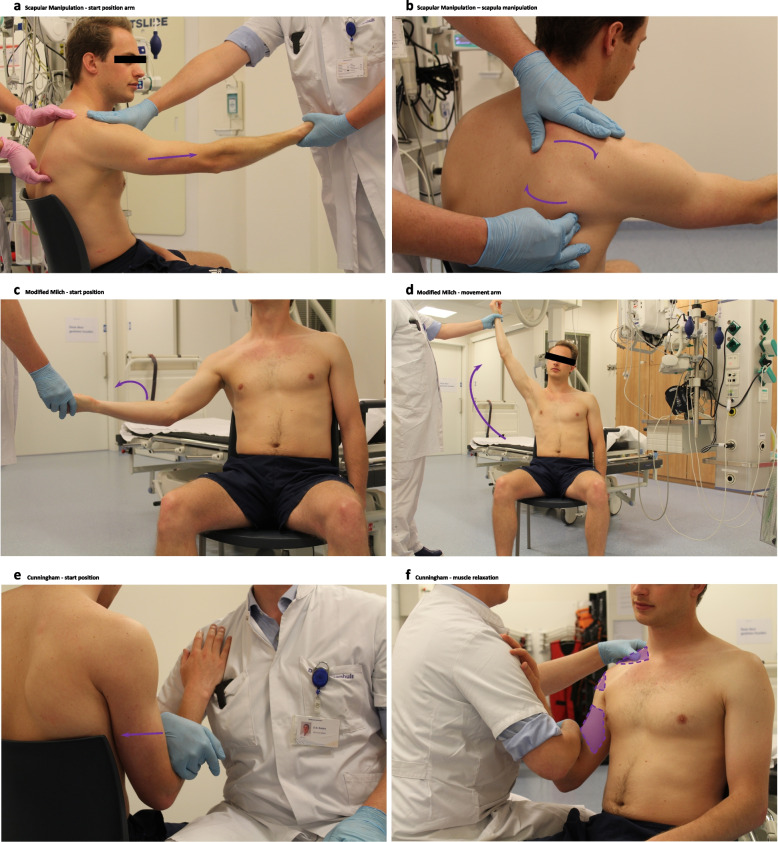
Description of reduction techniques: The pictures were taken at the emergency department of the Diakonessenhuis by the authors from a volunteer. Scapular manipulation. *How to perform*. Seated scapular manipulation allows the patient to remain seated upright. Facing the patient, a physician or assistant grasps the wrist of the patient’s affected side and slowly raises this to the horizontal plane and let the palm of the hand face upwards (exorotation) (Fig. 1a). Firm, but gentle, forward traction is applied with counterbalancing provided by placing the palm of the extended free arm over the patient’s midclavicular region. The force required in applying this traction is not great. Once gentle traction is applied, a second physician or assistant manipulates the scapula by applying constant pressure on the abducted inferior tip of the scapula to the medial, while holding the upper part of the scapula and putting pressure on this to the lateral. This allows the abducted inferior tip of the scapula to be rotated bringing the scapular neck and glenoid fossa into the correct alignment (Fig. 1b). It sometimes takes some time (minutes) before the muscles relax and the scapula moves. The shoulder is reduced when the scapula moves and the assistant feels the humeral head moving back in anatomical position [15]. Modified Milch. *How to perform*. Dislocated shoulder of the seated patient is positioned in an analgesic position by externally rotating the extended arm and slightly abducting anterolateral. This will decrease tension on m. infraspinatus, m. terres minor, and m. supraspinatus (Fig. 1c). Gently abduct the exorotated arm until reduction is achieved, often around 140–160° (Fig. 1d). Placing a thumb in the armpit can prevent the humeral head from sliding medially. No traction is used, but the arm is kept on length by the physician. After completing the 180° abduction, the arm is moved back in front of the patient to a neutral position. The procedure is painless, but sometimes gives discomfort when the humeral head is moving back in place. If there is pain while abducting the arm the speed of movement should be slowed down and/or a more anterior or posterior trajectory of the abduction can be followed [9, 16]. Cunningham. *How to perform*. The patient sits upright against a hard surface (i.e., chair of upright bed), the affected arm adducted to the body and the elbow fully flexed. The operator kneels/sits next to the patient and places his wrist onto the patient’s forearm (no pressure, this adds discomfort to the patient), the patient’s hand resting on the operator’s shoulder (Fig. 1e). It is important to keep the injured arm close to the body of the patient and flex their elbow to relax the bicep muscle. The patient is asked to shrug the shoulders superiorly and posteriorly, which “squares off” the angle of the shoulder (reducing scapular anteversion and the static obstruction of the glenoid rim). Start with the trapezius and deltoid muscles and afterwards move to the bicep muscle (Fig. 1f). Then, the biceps is massaged at a mid-humeral level to specifically relax the muscle (removing dynamic obstruction). The massage by the physician is not to relax the muscles, but to make the patient conscious of their muscle tension. Reduction is often after a couples of minutes and is expected when the shoulder contour has been restored [9]

In the “adduction” group, patients were randomized to one of three techniques: the Cunningham (CH), the modified Milch (MM), and the scapular manipulation technique (SMT). See Fig. 1 and the videos for a description of the techniques. There was a crossover to a pre-determined second technique after the attempt with the first allocated technique failed, if both techniques were unsuccessful the subsequent (third) technique used was left to the discretion of the treating physician. In the “no-adduction” group, patients were randomized to two techniques: the MM and the SMT. Criteria for crossover were the same. It was advised in the pre-trial training to try the Cunningham and the scapular manipulation technique for at least 10 min. If necessary the operator could perform a repositioning of the arm during the modified Milch technique.

To compare the techniques as reliably as possible, the study protocol explicitly advised against the use of pain medication during the process of shoulder reduction. Any medication given before arrival in the emergency department or during triage was recorded. If the study protocol was not successful and a third technique was used, the treating physician could use medication to his or her discretion, this was also recorded. After shoulder reduction, all patients received a radiograph to confirm reduction and the shoulder was immobilized in internal rotation with a sling. Regular follow-up in the outpatient clinic was scheduled in accordance with regular care.

### Training

All involved medical staff underwent training from the two lead investigators of the reduction techniques prior to the study, theoretical as well as a hands-on training. Additional information was also provided in the form of a pocket card and the URL of our YouTube channel (see link under videos). In all four participating centers, one of the investigators was available for questions if requested about the techniques and the study protocol.

### Patient and public involvement

In our work in the ED, we asked patients during their ED visit about their key points of attention in both the reduction as the process of the ED visit. We have based our research questions on those stories.. Because most patients have had a shoulder dislocation only once or a limited number of times, we found it difficult to engage them in research. And we did not involve patients or the public in the development of the protocol or analysis of the results.

### Primary outcomes

The primary outcomes were the length of stay at the emergency department, defined as the time between the moment of arrival at the emergency department until the patient was discharged, and the maximum levels of pain experienced by the patient during the reduction. The pain was measured using the numeric rating scale (NRS), which ranges from 0 to 10.

### Secondary outcomes

The secondary outcomes were the time needed for the reduction in minutes from the start until the end of reduction, the reduction success, effect of habitual dislocation on reduction success, the number of techniques used for reduction, use of analgesia or sedatives administered in the ED, and possible complications of the reduction (bony or neurovascular).

### Data collection

The physician responsible for study inclusion prospectively recorded pain scores, reduction time, reduction success, age, sex, dislocation side, dislocation time, previous dislocations, history, injury mechanism, any complications, reduction time, and neurovascular examination before and after reduction. In addition, the time required for actual reduction was recorded using a clock present in the treatment room, different timepoints of the ED stay (arrival, radiograph, reduction start, end and departure from the ED) and NRS were also noted during the reduction by the treating medical staff present at the reduction (see supplemental file 2 for all recorded parameters). All pre- and post-reduction radiographs were reviewed by the treating physician, the radiologist on call, and afterwards (blinded for the result of physician and radiologist) by the two main investigators (DB and MR) to assess for fractures.

### Statistical analysis

The sample size was calculated based on a clinically relevant difference in length of stay at the emergency department of 15 min between different techniques. The probability of a type 1 error (alpha) was set at 0.05 and with a power of 0.80; this led to a required total sample size of at least 62 in the no-adduction group and 123 in the “adduction” group, based on an assumed standard deviation of 20 min in both groups. Analyses were conducted according to the intention-to-treat principle. Continuous outcomes were compared using ANOVA (no-adduction group) or *t* test (adduction group) and binary outcomes using the chi-squared test. Only observed outcomes were included for analysis. A *p* value < 0.05 was considered statistically significant. All analyses were conducted using SPSS version 21 [17].

## Results

**Fig. 2:**
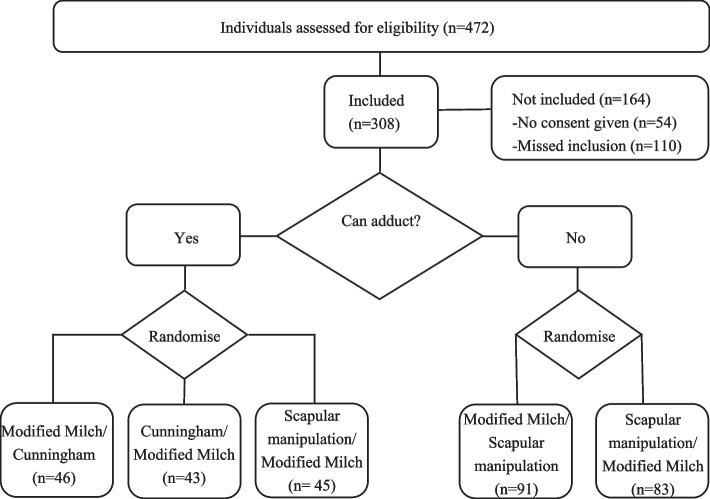
Randomization and inclusion schedule

Out of 472 eligible patients, 308 patients were included in the study. Of the 164 patients not included, 54 did not provide consent and 110 were excluded as the treating physician did not ask for consent. All randomized patients were included in the analyses (see Fig. 2). The shoulder reductions were carried out by 110 different professionals with varying experiences with the techniques. Of the included patients, 31% were treated by an emergency physician, 66% by an emergency medicine resident, and 3% by a nurse practitioner. These percentages were similar across the different treatment arms (see Supplementary Table 1 and Supplementary Table 2).

### Adduction group

The baseline characteristics of the 134 patients included in the adduction group are presented in Table 1. Their mean age was 50 years (SD 22), 50 patients were female (37%), and the most frequent cause of shoulder dislocation was a fall (*n* = 67 cases, 50%) (see Table 1).

**Table 1:** Characteristic of patients with anterior shoulder dislocation in the “adduction group” of the BRASD trial

Variable	Modified Milch/Cunningham	Cunningham/modified Milch	SMT/modified Milch
Number of patients	46	43	45
Mean age (SD)	48 (21)	51 (21)	51 (24)
Mean age men (SD)	38.9 (19.0)	42.8 (20.7)	43.3 (20.8)
Mean age women (SD)	64.3 (16.1)	64.4 (14.7)	63.2 (24.3)
Gender			
Men (%)	30 (65)	26 (60)	28 (62)
Female (%)	16 (35)	17 (40)	17 (38)
Dominant arm	*N* = 44	*N* = 43	*N* = 43
Right (%)	38 (83)	39 (90)	38 (84)
Left (%)	6 (17)	4 (10)	5 (16)
Dislocation in the dominant arm	*N* = 44	*N* = 43	*N* = 43
No (%)	16 (35)	20 (47)	20 (44)
Yes (%)	28 (65)	23 (53)	23 (56)
Trauma mechanism			
Sport	20	6	10
Seizure	1	2	3
Fall	16	27	24
Traffic accident	-	-	-
Other cause	9	8	8
NRS at arrival (SD)	7.4 (1.8)	7.3 (2.3)	7.2 (2.0)
Type of practitioner			
ED physician	9	17	18
Resident	35	26	26
Nurse practitioner	2	0	1
Medication used			
No	10	12	9
Oral medication	25	19	25
Intra-articular	0	0	0
IV medication	20	25	22
Fracture (pre-reduction)^a^	*N* = 38	*N* = 38	*N* = 43
No	29	25	25
Greater tuberosity	1	2	1
Bony Bankart lesion	4	8	9
Hill Sachs lesion	4	3	8
First-time dislocations (%)	28 (61)	30 (70)	29 (64)

^a^Numbers are different because of missing X-ray

#### Primary outcomes

The mean LOS at the emergency department for the adduction group was 120 min (SD 68 min). The mean total LOS at the ED was similar across treatment arms: in the MM/CH arm 110 min (SD 61 min), the CH/MM arm 125 min (SD 75 min), and the SMT/MM arm, it was 126 min (SD 69 min); *p* = 0.45.

There was no difference between the treatment arms regarding the maximum level of pain experienced by patients during reduction with the first technique (Table 2). The mean maximum level of pain experienced was 5.6 (SD 2.8) in the MM arm, 5.0 (SD 2.9) in the CH arm, and 6.1 (SD 2.8) in the SMT arm (*p* = 0.21 for comparison between treatment arms).

**Table 2:** Outcomes of patients with anterior shoulder dislocation in the adduction group of the BRASD trial

	Modified Milch/Cunningham	Cunningham/modified Milch	SMT/modified Milch	*p* value
	*n* = 46	*n* = 43	*n* = 45	
**Primary outcomes**				
Time total length of stay (minutes, SD)	110 (61)	125 (75)	126 (69)	0.45
NRS first technique during the reduction	5.6 (2.8)	5.0 (2.9)	6.1 (2.8)	0.21
Maximum NRS during reduction within protocol (SD)	5.7 (2.9)	5.8 (3.0)	6.4 (2.5)	0.41
**Secondary outcome**				
Time reduction first technique successful (minutes, SD)	8 (11)	3 (2)	3 (3)	0.14
Time start reduction until end reduction; all reductions (minutes, SD)	20 (27)	29 (50)	21 (26)	0.43
Reduction success with the first technique	24 (52%)	10 (23%)	15 (33%)	0.016
Reduction success within protocol (%)	28 (61%)	27 (63%%)	29 (64%)	0.94
Reduction success without sedation in the ED	43 (94%)	38 (88%)	38 (84%)	0.39
Reduction success in the ED	46 (100%)	43 (100%)	45 (100%)	-
Reduction success first technique with first-time dislocation	14 (50%)	6 (20%)	10 (35%)	0.024
Reduction success first technique with recurrent dislocation	10 (55%)	4 (31%)	5 (31%)	0.36

#### Secondary outcomes

Overall, the mean time from the start of the reduction until the end of the reduction was 23 min (SD 35), without differences between the treatment arms (Table 2).

The overall success of reduction within the protocol (the two techniques combined) was 63%, with no relevant difference in the treatment arms. There is a relevant difference between treatment arms in first technique success: MM was successful in the first attempt in 52% of patients, while for CH and SMT, this was 23% and 33%, respectively (*p* = 0.016). Ultimately, with the use of PSA, all patients could be treated in the emergency department and no patient required neuromuscular blocking agents or general anesthesia for the reduction. No complications were documented for any of the reduction techniques.

### No-adduction group

The baseline characteristics of the 174 patients included in the no-adduction group are presented in Table 2. Their average age was 43 years (SD 19), 41 patients were women (24%), and the most frequent cause was a fall (*n* = 78 cases, 45%) (see Table 3).

**Table 3:** Characteristic of patients with anterior shoulder dislocation in the no-adduction group of the BRASD trial

Variable	Modified Milch/SMT	SMT/modified Milch
Number of patients	91	83
Mean age (SD)	44 (20)	42 (19)
Mean age men (SD)	38.5 (16.6)	37.7 (16.5)
Mean age women (SD)	61.8 (19.9)	54.6 (19.4)
Gender		
Men (%)	69 (76)	64 (77)
Female (%)	22 (24)	19 (23)
Dominant arm	*N* = 90	*N* = 80
Right (%)	78 (86)	74 (89)
Left (%)	12 (14)	6 (11)
Dislocation in the dominant arm	*N* = 90	*N* = 80
No (%)	40 (44)	37 (46)
Yes (%)	50 (56)	43 (54)
Trauma mechanism		
Sport	23	27
Seizure	2	2
Fall	42	36
Traffic	4	1
Others	20	17
NRS at arrival (SD)	7.2 (2.6)	7.5 (2.0)
Type of practitioner	*N* = 90	*N* = 83
ED physician	32	19
Resident	55	61
Nurse practitioner	3	3
Medication used		
No	19	17
Oral medication	50	41
Intra-articular	1	1
IV medication	42	49
Fracture (pre-reduction)^a^	*N* = 76	*N* = 74
No	50	49
Greater tuberosity	6	6
Bony Bankart lesion	8	11
Hill Sachs lesion	12	8
First-time dislocations (%)	53 (58%)	35 (42%)

^a^Numbers are different because of missing X-ray

#### Primary outcome

The mean total LOS at the emergency department for the no-adduction group was 109 min (SD 65 min), in the MM/SMT arm 114 min (SD 60 min), and in the SMT/MM arm 104 min (SD 64 min); *p* = 0.30.

There was no difference between the treatment arms in this group regarding the maximum level of pain experienced by patients during reduction with the first technique (Table 4). The mean maximum level of pain experienced was 6.4 (SD 2.8) in the MM treatment arm and 6.3 (SD 2.9) in the SMT treatment arm (*p* = 0.91 for comparison between treatment arms).

**Table 4:** Outcomes of patients with anterior shoulder dislocation in the no-adduction group of the BRASD trial

	Modified Milch/SMT	SMT/modified Milch	*P* value
	*n* = 91	*n* = 83	
**Primary outcome**			
Time total length of stay (minutes, SD)	114 (60)	104 (64)	0.30
NRS first technique during reduction (SD)	6.1(2.9)	*N* = 82 5.9 (2.8)	0.68
Maximum NRS during reduction within protocol (SD)	*N* = 90 6.4 (2.8)	*N* = 82 6.3 (2.9)	0.91
**Secondary outcome**			
Time reduction first technique successful (minutes, SD)	5 (4)	3 (3)	0.003
Time start reduction until end reduction; all reductions (minutes, SD)	25 (39)	17 (21)	0.93
Reduction success with the first technique	47 (51%)	30 (36%)	0.040
Reduction success within protocol	60(66%)	54 (65%)	0.90
Reduction success without sedation in the ED	75 (82%)	71 (86%)	0.58
Reduction success in the ED	89 (98%)	81 (98%)	0.93
Reduction success first technique with first-time dislocation	27 (51%)	14 (30%)	0.032
Reduction success first technique with recurrent dislocation	20 (53%)	16 (46%)	0.56
Success first technique in age groups:			
18–45	34 (65%)	23 (43%)	0.024
46–older	13 (33%)	7 (23%)	0.36 *p* value for interaction: 0.55

#### Secondary outcome

Overall, the mean time from the start of the reduction until the end of the reduction was 20 min (SD 15 min). The difference between the treatment arms was not significant: in the MM/SMT arm 25 min (SD 39 min and in the SMT/MM arm 17 min (SD 21 min; *p* = 0.93).The overall success of reduction within the protocol (both techniques combined) was 66%, with no relevant difference in the treatment arms. Relevant differences were observed between treatment arms regarding the success rate of the first reduction technique: MM was successful in 51% of patients and SMT in 36% of patients (*p* = 0.040).

This reduction success rates increased to 98% by use of PSA in the ED. In both study arms, two patients required general anesthesia and muscular blocking agents in the operating room for reduction. No complications were recorded in any of the techniques.

## Discussion

In this randomized trial, no differences were found regarding the length of stay in the emergency department during the reduction process, regardless of the applied shoulder biomechanical reduction technique or the possibility to adduct the arm. Also, neither in the adduction nor in the no-adduction group differences were observed concerning the maximum pain endured during the reduction. With regard to the reduction time, no differences were observed in the adduction group, yet a difference was observed in favor of the scapular manipulation technique among patients who could not perform adduction of the injured arm.

In both groups, a larger initial success rate was observed in the modified Milch treatment arm compared to the other reduction techniques. Ultimately, the success rate in the ED of both groups is very high, 100% and 98% for the adduction and no-adduction groups, respectively.

The total LOS in the ED does not seem to be influenced by the technique used. This is probably because LOS is influenced by multiple factors such as time to triage, the treating physician and staff, waiting time for the pre- and post-reduction radiograph, and time until discharge [18]. The LOS is also influenced by the success rate of the first technique. No prior studies are available for comparison.

Pain experienced during the reduction process was lower in both groups compared to pain at the time of emergency department arrival. This is in line with another study on biomechanical reduction techniques [6]. The maximum pain during the first reduction attempt corresponds with Amar et al., but are higher than in 2 other studies [5, 6, 12]. The reason could be the timing of the measurement of the pain scores. Amar et al. analyzed pain scores at several moments in time too, yet the two other studies only determined the pain score during the actual reduction, possibly making it more difficult for the patient to indicate the changes in the experienced pain.

The reported reduction time of a successful reduction using the first allocated technique is similar to what is reported in the literature on biomechanical techniques varying from 130 to 281 s [5, 6, 12, 13]. The total LOS does not seem to vary between the arms in our study. We think that the total time needed for the reduction in our study is influenced more by providers switching back and forth between different maneuvers than by the techniques used. Factors for this extra time can be that additional assistance might be required or the patient requires additional explanation. Also, the need for PSA can increase the time needed for the reduction time [18].

Studies with biomechanical techniques showed a first technique success between 69 and 89% [5, 6, 12, 13]. To our knowledge, there are no previous trials directly comparing SMT or Cunningham which makes it harder to compare the first technique’s success rate. In the present study, we observed lower first-reduction success rates compared to previous studies assessing the modified Milch technique [13]. This might be influenced by the wide range of experience of the treating clinicians in this study. Another explanation might be that we advised treating clinicians to take a minimum of 10 min per technique, to ensure the required adequate muscle relaxation. Perhaps this time frame was too short since eventually there was an almost 100% reduction success without the use of IAL or PSA.

Our results demonstrated that the modified Milch technique had the highest first-technique success rate in both groups. This could be due to the fact that the technique is easy to learn, with few pitfalls in execution. Perhaps the effect of the technique is enhanced by the patients’ awareness of the actual reduction process. Both in SMT and Cunningham, we considered that the mechanism of relaxation is less obvious to the patient and so they have less positive feedback on the relaxation of the muscles. Also, the SMT is less desirable because it requires two people to perform.

The maximum pain during the first reduction attempt seems to be in line with other studies, these studies seem to have a higher NRS if multiple pain scores are done in contrast with only one during the reduction [5, 12, 13]. We found comparable pain scores with the study doing multiple pain scores. This might be of importance when analyzing patients’ pain experiences in future studies.

This study has several strengths; it is a randomized multicenter trial, equally distributed across the participating centers. In addition, it is applicable to the average day-to-day emergency department setting. A large group of medical staff was involved, often with little experience, and the study included a heterogeneous population of patients. Attention was also directed to (limiting) medication use, which we think is a confounder in reduction studies. In a recently performed survey, we showed that ED providers use traction techniques and biomechanical techniques almost as often as their first technique, this study could warrant the use of biomechanical techniques even further [11].

This study also has limitations. First, patients may have wrongfully entered the no-adduction group, for example, due to the inexperience of medical staff who had to classify participants. Proper judgment of the ability to adduct the arm in a painful situation of a shoulder dislocation requires experience. Second, despite the training of the medical staff, it is possible that there has been variation in the implementation of the techniques. Third, due to a large number of treating clinicians, including residents, and the relatively small number of reductions per physician, there could have been knowledge decay of the techniques. Fourth, traction-counter traction or leverage techniques were not included in our study for comparison as, in our experience, patients require sedation for proper reduction with these techniques. Fifth, the power calculation of our primary outcome LOS was based on the assumption that the standard deviation (SD) of LOS would be approximately 20 min, while this turned out to be 75 min. The larger SD makes our study (in hindsight) underpowered to detect a difference in LOS of 15 min between the techniques. Finally, after a review of the sealed envelopes at the end of the study, there were six envelopes missing in total, four in the adduction and two in the no-adduction group. This is less than 2% of the total inclusions, and therefore, we do not think this has substantially influenced the outcome of the study.

Ultimately, one might conclude that LOS is not the ideal primary outcome measure to analyze the reduction success. The influence of the other factors turned out to be much greater than the actual reduction time. In future research, reduction success is the better choice as the primary endpoint.

Future research should focus on several areas: (1) the influence of muscle tension and muscle group relaxation on successful reduction, (2) the effect of the learning curve of the biomechanical techniques on reduction time and success, and (3) it would be interesting to see if there are techniques that have a faster turnaround time and higher success rate in older patients.

## Conclusion

This is the first randomized study to compare multiple biomechanical shoulder reduction techniques with regard to the patient length of stay in the emergency department and pain experience, minimizing and recording the impact of confounders, especially medication use. The different techniques did not appear to influence the total length of stay in the emergency department or the reduction time. There was also no difference in perceived pain. This study demonstrates that a near 100% reduction success in the ED is possible when using a combination of biomechanical shoulder reduction techniques, encouraging every ED physician to acquire these techniques. We recommend starting a reduction with the modified Milch technique.

## Supplementary Information

**Additional file 1.** Consort checklist BRASD.

**Additional file 2.** Recorded data.

**Additional file 3: **Table S1. Supplement group characteristics and results: ‘Adduction group’.

**Additional file 4:** Table S2. Supplement group characteristics and results: ‘No-Adduction group’.

## Data Availability

The datasets used and/or analyzed during the current study are available from the corresponding author upon reasonable request. Videos: 1.Cunningham: https://youtu.be/6TF3h3RNS0M 2.Modified Milch: https://youtu.be/yOm1bF-U9Q8 3.Scapular manipulation technique https://youtu.be/Cig7XRH8cZs 4.YouTube channel BRASD-trial playlist https://www.youtube.com/playlist?list=PLE9SsnaLVuUIHFDxaos05Hgsb0cJ3Yt0P

## References

[1] Baden DN, Roetman MH, Boeije T, Roodheuvel F, Mullaart-Jansen N, Peeters S (2017). Biomechanical reposition techniques in anterior shoulder dislocation: a randomised multicentre clinical trial -the BRASD-trial protocol. BMJ Open.

[2] Alkaduhimi H, van der Linde JA, Willigenburg NW, van Deurzen DFP, van den Bekerom MPJ (2017). A systematic comparison of the closed shoulder reduction techniques. Arch Orthop Trauma Surg.

[3] Wakai A, O’Sullivan R, McCabe A (2011). Intra-articular lignocaine versus intravenous analgesia with or without sedation for manual reduction of acute anterior shoulder dislocation in adults. Cochrane Database Syst Rev.

[4] Jiang N, Hu YJ, Zhang KR, Zhang S, Bin Y (2014). Intra-articular lidocaine versus intravenous analgesia and sedation for manual closed reduction of acute anterior shoulder dislocation: an updated meta-analysis. J Clin Anesth.

[5] Sayegh FE, Kenanidis EI, Papavasiliou KA, Potoupnis ME, Kirkos JM, Kapetanos GA (2009). Reduction of acute anterior dislocations: a prospective randomized study comparing a new technique with the hippocratic and Kocher methods. J Bone Jt Surg.

[6] Amar E, Maman E, Khashan M, Kauffman E, Rath E, Chechik O (2012). Milch versus Stimson technique for nonsedated reduction of anterior shoulder dislocation: a prospective randomized trial and analysis of factors affecting success. J Shoulder Elb Surg.

[7] Mattick A, Wyatt JP (2000). From Hippocrates to the Eskimo–a history of techniques used to reduce anterior dislocation of the shoulder. J R Coll Surg Edinb.

[8] Riebel GD, McCabe JB (1991). Anterior shoulder dislocation: a review of reduction techniques. Am J Emerg Med.

[9] Cunningham NJ (2005). Techniques for reduction of anteroinferior shoulder dislocation. Emerg Med Australas.

[10] Alkaduhimi H, van der Linde JA, Flipsen M, van Deurzen DFP, van den Bekerom MPJ (2016). A systematic and technical guide on how to reduce a shoulder dislocation. Turkish J Emerg Med Elsevier Ltd.

[11] Baden DN, Roetman MH, Boeije T, Mullaart-Jansen N, Burg MD (2020). A survey of emergency providers regarding the current management of anterior shoulder dislocations. J Emergencies, Trauma Shock.

[12] Maity A, Roy DS, Mondal BC (2012). A prospective randomised clinical trial comparing FARES method with the Eachempati external rotation method for reduction of acute anterior dislocation of shoulder. Injury.

[13] Sapkota K, Shrestha B, Onta PR, Thapa P (2015). Comparison between external rotation method and milch method for reduction of acute anterior dislocation of shoulder. J Clin Diagnostic Res.

[14] Hovelius L, Augustini BG, Fredin H, Johansson O, Norlin R, Thorling J (1996). Primary anterior dislocation of the shoulder in young patients. J bone Jt Surg.

[15] McNamara RM (1993). Reduction of anterior shoulder dislocations by scapular manipulation. Ann Emerg Med.

[16] Milch H (1949). The treatment of recent dislocations and fracture-dislocations of the shoulder. J Bone Joint Surg Am.

[17] IBM Corp. Released 2012. IBM SPSS Statistics for Windows, Version 21.0. Armonk, NY: IBM Corp.

[18] Schuur D, Baden DN, Roetman M, Boeije T, Burg M, Mullaart-Jansen N (2020). Which factors influence the ED length-of-stay after anterior shoulder dislocations: a retrospective chart review in 716 cases. BMC Emerg Med.

