# Supplementary material for: BRASD trial: biomechanical reposition techniques in anterior shoulder dislocation—a randomized multicenter clinical trial
Source: Int J Emerg Med. 2023 Feb 24;16:14. doi: 10.1186/s12245-023-00480-6 (PMC9951479; doi:10.1186/s12245-023-00480-6)
Supplement: Supplementary file 2 — Additional file 2. Recorded data. [file 12245_2023_480_MOESM2_ESM.pdf]

Supplement 1 Recorded data

Age

Sex

Function treating physician (Emergency physicians, emergency resident, nurse practitioner)

Treatment date

Arrival time in the ED

Dislocation side

Means of arrival

Date and time of dislocation

Trauma mechanism

Number of previous dislocations

Number of reduction attempts before hospital arrival

Dominant arm

NRS at arrival

Last meal

Medication before hospital arrival or in triage

Fracture on X-ray before reduction

Position of humeral head before reduction

1st Reduction start time

1st Reduction end time

NRS before 1st reduction

NRS during 1st reduction

NRS after 1st reduction

2nd Reduction start time of applicable

2nd Reduction end time of applicable

NRS before 2nd reduction

NRS during 2nd reduction

NRS after 2nd reduction

3th Reduction start time of applicable

3th Reduction end time of applicable

Medication used during reduction

Sedation

Reduction in the operating room

Neurovascular status before reduction

Neurovascular status after reduction

Time of x-ray before reduction

Time of x-ray after reduction

New fracture on X-ray after reduction

Discharge time of ED
