# Supplementary material for: BRASD trial: biomechanical reposition techniques in anterior shoulder dislocation—a randomized multicenter clinical trial
Source: Int J Emerg Med. 2023 Feb 24;16:14. doi: 10.1186/s12245-023-00480-6 (PMC9951479; doi:10.1186/s12245-023-00480-6)
Supplement: Supplementary file 3 — Additional file 3: Table S1. Supplement group characteristics and results: ‘Adduction group’. [file 12245_2023_480_MOESM3_ESM.pdf]

Supplementary Table 1 Supplement group characteristics and results – 'Adduction group'

	Modified Milch/Cunningham	Cunningham/Modified Milch	SMT/Modified Milch	P-value
Number of patients	46	43	45	
**Characteristics**				
Function treating professional:				
Emergency physician	9	17	18	
Resident	35	26	26	0.14
Nurse practitioner	2	0	1	
Pre-enrolment medication use:				
No	10	12	9	0.85
Oral medication	25	19	25	0.51
Intra-articular	0	0	0	-
IV Opioids/Benzodiazepines	20	25	22	0.38
Medication use during reduction:				
No	37	30	35	0.47
Oral medication	1	1	1	1.0
Intra-articular	2	8	3	0.05
IV Opioids	7	10	6	0.43
Pre-reduction fractures:	N=38	N=38	N=43	
None	29	25	25	
Tuberculum majus	1	2	1	0.51
Bankart	4	8	9	
Hill Sachs	4	3	8	
**Results**				
NRS first technique pre-reduction	6.37 (2.1)	6.3 (2.5)	6.47 (2.32)	0.94
NRS first technique during reduction	5.57 (2.8)	5.0 (2.9)	6.07 (2.8)	0.21
NRS first technique post-reduction	4.09 (3.2)	4.63 (2.9)	N=44<br>4.8 (2.98)	0.51
Number of techniques used:				
1	10 (55%)	4 (31%)	5 (31%)	
2	3 (17%)	6 (46%)	7 (44%)	0.36
3+	5 (28%)	3 (23%)	4 (25%)	
Number of techniques used non-habitual				
1	14 (50%)	6 (20%)	10 (35%)	
2	1 (4%)	11 (37%)	7 (24%)	0.024
3+	13 (47%)	13 (43%)	12 (41%)	
Number of techniques used habitual				
1				

2<br>3+	10 (55%)<br>3 (17%)<br>5 (28%)	4 (31%)<br>6 (46%)<br>3 (23%)	5 (31%)<br>7 (44%)<br>4 (25%)	0.36
Type of third techniques used:<br>Traction<br>Leverage<br>Biomechanical	 12 (26%)<br>3 (7%)<br>3 (7%)	 8 (19%)<br>5 (12%)<br>3 (7%)	 10 (22%)<br>3 (7%)<br>3 (7%)	0.85
