# Supplementary material for: BRASD trial: biomechanical reposition techniques in anterior shoulder dislocation—a randomized multicenter clinical trial
Source: Int J Emerg Med. 2023 Feb 24;16:14. doi: 10.1186/s12245-023-00480-6 (PMC9951479; doi:10.1186/s12245-023-00480-6)
Supplement: Supplementary file 4 — Additional file 4: Table S2. Supplement group characteristics and results: ‘No-Adduction group’. [file 12245_2023_480_MOESM4_ESM.pdf]

Supplementary Table 2 Supplement group characteristics and results – 'No-Adduction group'

Variable	Modified Milch/SMT	SMT/Modified Milch	P-value
Number of patients	90	83	
**Characteristics**			
Function treating professional:			
Emergency physician	32	19	
Resident	55	61	0.19
Nurse practitioner	3	3	
Pre-enrolment medication use:			
No	19	17	0.95
Oral medication	50	41	0.46
Intra-articular	1	1	0.95
IV Opioids/Benzodiazepines	42	49	0.09
Medication use during reduction:			
No	74	71	0.46
Oral medication	1	0	0.34
Intra-articular	7	4	0.44
IV Opioids	11	9	0.80
Pre-reduction fractures:	N=76	N=74	
None	50	49	
Tuberculum majus	6	6	0,74
Bankart	8	11	
Hill Sachs	12	8	
**Results**			
NRS first technique pre-reduction (SD)	6.72 (2.51)	6.69 (2.52)	0.78
NRS first technique during reduction (SD)	6.09(2.91)	N=82<br>5.89 (2.82)	0.68
NRS first technique post-reduction (SD)	4.40 (3.31)	N=80<br>4.89 (3.19)	0.52
Number of techniques used:			
1	47 (51%)	30 (36%)	
2	13 (14%)	24 (29%)	0.034
3+	31 (34%)	29 (35%)	
Number of techniques used non-habitual			
1	27 (51%)	14 (30%)	
2	9 (17%)	12 (25%)	0.10
3+	17 (32%)	21 (45%)	

Number of techniques used habitual			
1			
2	20 (53%)	16 (46%)	
3+	4 (10%)	12 (34%)	0.036
	14 (37%)	7 (20%)	
Type of third techniques used:			
Traction	15 (17%)	16 (19%)	
Leverage	5 (6%)	3 (4%)	0.77
Biomechanical	10 (11%)	9 (11%)	
